# Effectiveness of a healthcare retreat for male employees with cardiovascular risk factors

**DOI:** 10.1016/j.pmedr.2018.12.005

**Published:** 2018-12-18

**Authors:** Keiichi Matsuzaki, Shotaro Taniguchi, Kana Inoue, Takashi Kawamura

**Affiliations:** a Kyoto University Health Service, Yoshida-Honmachi, Sakyo-ku, Kyoto 606-8501, Japan; b Laboratory and Vascular Medicine, Kagoshima University Graduate School of Medical and Dental Sciences, 8-35-1, Sakuragaoka, Kagoshima 890-8520, Japan; c Miyazaki Prefectural Health Foundation, 1-1-2, Kirishima, Miyazaki City, Miyazaki 880-0032, Japan

**Keywords:** Lifestyle modification, Obesity, Cardiovascular risk factors, Healthcare retreat

## Abstract

Lifestyle modification is the primary treatment strategy for obesity, hypertension, dyslipidemia, and hyperglycemia. Recently, the Japanese government designed a healthcare retreat program for persons with cardiovascular risk factors. However, the structure and effectiveness of this program has not been fully discussed.

Employees of a company group with obesity and one or more other cardiovascular risk factors were enrolled in the study. The participants in the three-day retreat program were compared with those receiving a brochure-based advice for their subsequent changes in the annual health check-up data using the propensity score matching method.

Among the 415 eligible employees, 220 underwent the intensive program and 195 received a brochure-based advice. In the propensity score-matched subjects, reduction in body weight (2.7 kg vs. 0.99 kg, p < 0.01), waist circumference (3.5 cm vs. 1.5 cm, p < 0.01), and non-HDL cholesterol (8.8 mg/dl vs. 1.3 mg/dl, p = 0.05) were significantly greater in the intensive care group one year later. The superiority of the intensive program, however, was gradually attenuted for subsequent two years later.

This healthcare retreat with counseling and training program would improve body weight and waist circumference for one to two subsequent years.

## Highlights

• Lifestyle modification is the primary treatment strategy for cardiovascular disease.
• Based on the trans-theoretical model, healthcare retreat program was established.
• Mean 2.7 kg reduction in body weight after 1 year in healthcare retreat group
• Our healthcare retreat program may supersede conventional approach.

## 1 Introduction

Obesity, hypertension, dyslipidemia, and hyperglycemia are the well-known risk factors for cardiovascular diseases in economically advanced countries (Hunt et al., 2004; Malik et al., 2004), including Japan (Takeuchi et al., 2005). These preclinical pathologies often begin with accumulated visceral fat and subsequent insulin resistance derived from unhealthy lifestyles in addition to one's genetic predisposition. The primary strategy for visceral fat reduction is lifestyle modification (Alberti et al., 2006) ([Definition and the diagnostic standard for metabolic syndrome—Committee to Evaluate Diagnostic Standards for Metabolic Syndrome], 2005). Previous studies have confirmed that lifestyle modification programs, including both improved dietary habits and increased physical activity levels, reduce visceral fat deposits (Sacks et al., 2009) (Vissers et al., 2013) (Nazare et al., 2013). Persons with much visceral fat, therefore, can prevent subsequent diseases through lifestyle modification.

The Ministry of Health, Labor and Welfare of Japan (MHLW) has mandated health insurers to perform annual health check-ups measuring their adiposity, blood pressure, lipid profile, and glycemic status for insured persons aged 40 years or more. Since 2008, the MHLW further forced health insurer to take the 6-month specified formula of health counseling performed by physicians, public health nurses, and registered dieticians for persons having obesity and two or more of either hypertension, dyslipidemia, glucose intolerance, or smoking. Although multiple Japanese researchers have reported improved measurements for the aforementioned risk factors, only 30% of the counselees could attain >3% weight reduction. Additionally, the maintenance of weight loss has been poorly discussed and health professionals worry about the potential for short-term rebound in body weight and other risk factors.

In 2015, the MHLW proposed an retreat type lifestyle counseling and training, performed jointly by physicians, public health nurses, registered dietitians, health and physical fitness programmers, and various other therapists, and subsidized the forerunners of such programs. The healthcare retreat program is based on the trans-theoretical model (Prochaska and Velicer, 1997) and leads to health behavior change. However, in practice, the structure of healthcare retreat has not been established. Therefore, the development of counseling methods with definite and durable effects is a matter of urgency. Since 1985, the Sunstar Group has provided a three-day healthcare retreat program at the corporate health gymnasium for their employees with cardiovascular risk factors. The program contains counseling for lifestyle modification, lectures focusing on diet and physical exercise, and guidance on physical activities. To date, a total of 6500 employees have attended this program. This study aimed to evaluate the effectiveness of the program and its sustainability.

## 2 Methods

### 2.1 Annual health check-up

This study was conducted among approximately 1500 employees of the Sunstar Group (Sunstar Inc., Sunstar Engineering Inc., etc.) located in Japan. The Industrial Safety and Health Act of Japan requires employers to provide annual health check-ups for employees living in Japan. The Sunstar Group carries out the health check-ups in February and March every year. A health check-up consists of anthropometry, laboratory tests including urine and blood tests, and a self-administrated questionnaire about disease history, family history, and lifestyle.

Height and weight were simultaneously measured with a scale (Ueda Avance, Osaka, Japan). Body mass index (BMI) was calculated as body weight (kg) divided by height (m) squared. Waist circumference was measured at the level of the umbilicus in one's standing position using a tape measure. Systolic and diastolic blood pressure (SBP and DBP, respectively) was measured by an automatic sphygmomanometer (Elk Corporation, Osaka, Japan). Blood tests were performed to analyze the concentration of total cholesterol, high-density lipoprotein (HDL) cholesterol, low-density lipoprotein (LDL) cholesterol, triglyceride (TG), fasting plasma glucose (FPG), and hemoglobin A1c (HbA1c) by the Japan Diabetes Society method (about 0.4% lower than the National Glyco-hemoglobin Standardization Program). Non-HDL cholesterol was calculated from total cholesterol and HDL cholesterol.

### 2.2 Healthcare retreat program

According to the national strategy, the healthcare personnel of the company group extracted the employees who underwent annual health check-up and met the below-mentioned “aggressive support” or “motivational support” criteria for metabolic syndrome, and invited to the three-day healthcare retreat program (intensive care) at the health gymnasium (Shin-Shin Kenko Dojo) between June and October right after the health checkup. The criteria for “aggressive support” and “motivational support” were as follows: waist circumference ≥85 cm or BMI ≥ 25, with at least one (“motivational support” level) or two (“aggressive support” level) of FPG ≥ 100 mg/dl or HbA1c ≥ 5.2%, HDL cholesterol <40 mg/dl or TG ≥ 150 mg/dl, SBP ≥ 130 mm Hg or DBP ≥ 85 mm Hg, and cullently smoking.

Participants received detailed information regarding their health check-up results at the beginning of the program and attended a series of lectures about metabolic syndrome, cardiovascular diseases, diet therapy, and exercise therapy. Unmilled brown rice and green vegetable smoothies were provided in meals, but animal products were not included. By such meals, total caloric energy consumption was limited to 1200 kcal per day. Physical exercise included aerobics, water aerobics, and walking at 3 metabolic equivalents (METs) for 2 h per day. At the end of the program, the participants decided and declared their own goals.

If an eligible employee could not attend the program, he or she received a brochure for general advice (usual care).

### 2.3 Study subjects and data collection

All the candidates for the three-day healthcare retreat program in 2007, 2008 or 2009 were enrolled in this study. Those who left the company group in the subsequent three years were excluded from the study. Then, the study subjects were classified into the intensive care group (participants in the three-day healthcare retreat program at the health gymnasium by 2010) or the usual care group (non-participants in the program during the study period).

The health check-up data at the baseline and one, two and three years later were collected for each study subject.

### 2.4 Statistical analysis

Changes in the measurements for each cardiovascular risk factor from baseline to one, two, and three years after the program were compared between the intensive care group and the usual care group. Statistical analyses were performed using Stata Version 15 (StataCorp, College Station, TX, USA). Normally distributed continuous variables were expressed with mean and standard deviations and compared using Student's *t*-test. Non-normally distributed continuous variables were expressed with medians and interquartile ranges and compared using the Mann–Whitney *U* test. Categorical variables were expressed as a number count with proportions and analyzed using the chi-square test or Fisher's exact test.

Using a logistic regression model, we calculated a propensity score for receiving (choosing to attend) the healthcare retreat. The variables included in the model (Austin, 2011) were age, height, weight, waist circumference, BMI, SBP, DBP, HbA1c, total cholesterol, HDL cholesterol, LDL cholesterol, TG, FPG, the number of the risk factors for metabolic syndrome, and the categories of the health counseling (aggressive or motivational) at baseline. One-to-one nearest neighbor matching between the intensive care group and the usual care group was performed with a caliper width of 0.2 of the standard deciation of the logit of the propensity score, permitting a maximum standardized mean difference of 0.10. All probability values were two-tailed, and all confidence intervals were compared at 95%.

### 2.5 Ethical issue

Individual informed consent was waived for the use of de-identified clinical data according to the national ethical guidelines of Japan. Instead, an opt-out approach was applied for this study. All personal identifiers were removed before the researchers obtained the dataset from the Sunstar Group. The Kyoto University Graduate School and Faculty of Medicine Ethics Committee approved the study protocol (registration number: R1121).

## 3 Results

Among 415 employees who met the eligibility criteria, 220 subjects received the three-day healthcare retreat program at the gymnasium (intensive care group) and 195 received only usual brochure-based advice (usual care group). Table 1 shows the baseline characteristics of the subjects. We extracted 190 subjects using the propensity score matching. Body weight (78.7 ± 10.1 kg vs. 74.3 ± 8.6 kg, p < 0.01), waist circumference (92.4 ± 6.9 cm vs. 90.2 ± 6.3 cm, p < 0.01), non-HDL cholesterol (166.0 ± 29.0 mg/dl vs. 159.8 ± 34.9 mg/dl, p = 0.05), and TG (168.0 ± 83.6 mg/dl vs. 138.7 ± 92.6 mg/dl, p < 0.01), were significantly greater in the intensive care group.

**Table 1:** Baseline characteristics in the intensive care group and the usual care group.

	Total (n = 415)	Intensive care (n = 220)	Usual care (n = 195)	p
Age, years (SD)	45.5 (8.7)	43.9 (8.2)	47.4 (8.8)	<0.01
Height, cm (SD)	171.7 (5.8)	172.3 (5.8)	171.0 (5.8)	0.03
Weight, kg (SD)	76.6 (9.7)	78.7 (10.1)	74.3 (8.6)	<0.01
Waist circumference, cm (SD)	91.4 (6.7)	92.4 (6.9)	90.2 (6.3)	<0.01
BMI	26.0 (3.0)	26.5 (3.1)	25.4 (2.7)	<0.01
Systolic blood pressure, mm Hg (SD)	127.7 (12.7)	127.4 (12.1)	128.2 (13.2)	0.52
Diastolic blood pressure, mm Hg (SD)	79.1 (9.8)	80.1 (9.7)	77.9 (9.8)	0.03
Total cholesterol, mg/dl (SD)	215.1 (31.5)	214.8 (28.2)	215.5 (34.9)	0.80
HDL cholesterol, mg/dl (SD)	52.0 (13.2)	48.7 (11.7)	55.8 (13.9)	<0.01
Non-HDL cholesterol, mg/dl (SD)	163.1 (32.0)	166.0 (29.0)	159.8 (34.9)	0.05
TG, mg/dl (SD)	154.3 (89.0)	168.0 (83.6)	138.7 (92.6)	<0.01
Fasting plasma glucose, mg/dl (SD)	104.0 (18.9)	101.1 (11.6)	107.3 (24.3)	<0.01
HbA1c (JDS),% (SD)	5.2 (0.8)	5.1 (0.4)	5.4 (1.1)	<0.01

Abbreviations: BMI, body mass index; TG, triglyceride; JDS, Japan Diabetes Society.

Table 2 shows the cardiovascular risk factor profiles in the propensity score-matched subjects at baseline and one, two and three years after the program. The baseline characteristics were almost identical in both groups for all items. Body weight was reduced by 2.7 kg in the intensive care group, while by 1.0 kg in the usual care group one year later (p < 0.01). Similarly, waist circumference was reduced by 3.5 cm and 1.5 cm in the intensive care and usual care groups, respectively (p < 0.01). The reduction in body weight and waist circumference was well preserved even two years later (2.8 kg and 3.8 cm, respectively) and three years later (2.4 kg and 3.3 cm, respectively) in the intensive care group. By contrast, body weight and waist circumference were gradually reduced (1.7 kg and 2.3 cm reduction, respectively, two years later and 1.7 kg and 2.6 cm reduction, respectively, three years later) in the usual care group. Therefore, intergroup differences in the reduction of body weight and waist circumference were attenuated year by year.

**Table 2:** Cardiovascular risk factor profiles at baseline, one, two, and three years after the program for the propensity score-matched* intensive care and usual care groups.

	Baseline			1 year later				
	Intensive care group (n = 95)	Usual care (n = 95)	p	Intensive care group (n = 95)		Usual care (n = 95)		p
				Measurement	Change	Measurement	Change	
Age (SD)	46.4 (8.4)	46.9 (8.9)	0.74	–	–	–	–	–
Weight, kg (SD)	74.7 (7.8)	74.5 (7.5)	0.86	71.9 (7.8)	−2.7 (3.4)	73.5(7.9)	−0.99 (2.9)	<0.01
Waist circumference, cm (SD)	90.4 (5.7)	90.2 (5.9)	0.73	86.9(6.0)	−3.5 (3.6)	88.7(6.7)	−1.5 (3.9)	<0.01
Systolic blood pressure, mm Hg (SD)	128.4 (13.4)	127.1 (12.0)	0.49	124.5 (14.8)	−3.9 (11.5)	125.7 (15.6)	−1.4 (12.9)	0.17
Diastolic blood pressure, mm Hg (SD)	78.2 (9.5)	78.0 (9.6)	0.88	78.9 (10.5)	0.65 (7.6)	79.8 (10.1)	1.8 (9.5)	0.36
Total cholesterol, mg/dl (SD)	213.9 (28.1)	217.7 (35.1)	0.40	208.0 (34.9)	−5.9 (25.4)	216.6 (37.3)	−1.1 (26.7)	0.21
HDL cholesterol, mg/dl (SD)	53.2 (13.4)	53.3 (13.7)	0.95	54.2 (12.8)	0.99 (6.4)	54.0 (13.3)	0.71 (9.0)	0.80
Non-HDL cholesterol, mg/dl (SD)	160.7 (29.2)	164.4 (33.7)	0.41	153.8 (35.1)	−8.8 (24.0)	162.6 (36.6)	−1.8 (24.8)	0.05
TG, mg/dl (SD)	143.8 (66.7)	145.0 (65.4)	0.90	116.8 (64.7)	−27.0 (65.1)	130.1 (64.0)	−14.9 (56.9)	0.18
Fasting plasma glucose, mg/dl (SD)	103.0 (13.0)	103.6 (15.9)	0.78	100.4 (12.1)	−2.7 (8.2)	103.5 (16.3)	−0.2 (11.0)	0.08
HbA1c (JDS),% (SD)	5.2 (0.5)	5.1 (0.6)	0.80	5.1 (0.4)	−0.05 (0.25)	5.1 (0.6)	0.1 (0.34)	0.18


	2 years later					3 years later				
	Intensive care group (n = 95)		Usual care (n = 95)		p	Intensive care group (n = 95)		Usual care (n = 95)		p
	Measurement	Change	Measurement	Change		Measurement	Change	Measurement	Change	
Age	–	–	–	–	–	–	–	–	–	–
Weight, kg (SD)	72.0 (8.1)	−2.8 (4.3)	72.6(8.1)	−1.7 (3.5)	0.07	72.6 (8.4)	−2.4(4.4)	73.0(7.6)	−1.7 (3.2)	0.27
Waist circumference, cm (SD)	86.6 (7.1)	−3.8 (4.7)	87.9(7.1)	−2.3 (3.9)	0.02	87.1 (7.2)	−3.3(4.8)	87.7 (6.9)	−2.6 (3.7)	0.24
Systolic blood pressure, mm Hg (SD)	124.0 (13.9)	−4.5 (12.6)	125.1 (14.5)	−2 (11.8)	0.17	125.4 (13.8)	−3.3(14.0)	123.0 (12.1)	−4.2 (10.8)	0.66
Diastolic blood pressure, mm Hg (SD)	78.8 (9.4)	0.45 (8.1)	79.8 (10.2)	1.8 (9.3)	0.31	78.8 (9.7)	0.51(9.0)	77.5 (8.9)	−0.96 (8.9)	0.28
Total cholesterol, mg/dl (SD)	209.5 (30.5)	−4.0 (23.7)	214.6 (31.2)	−2.8 (26.9)	0.74	213.8 (34.2)	0.10(25.0)	211.2 (30.9)	−5.7 (33.0)	0.44
HDL cholesterol, mg/dl (SD)	56.1 (15.4)	2.9 (8.9)	55.1 (12.9)	1.8 (9.0)	0.42	57.8 (14.6)	4.7(8.0)	56.0 (13.8)	2.6 (8.4)	0.10
Non-HDL cholesterol, mg/dl (SD)	153.3 (31.9)	−6.6 (25.1)	159.5 (29.8)	−4.6 (24.7)	0.59	156.0 (32.9)	−4.6(25.2)	155.2 (29.6)	−8.3 (31.2)	0.86
TG, mg/dl (SD)	118.7 (64.1)	−25.2 (61.0)	132.0 (74.0)	−13.2 (66.6)	0.20	119.7 (61.0)	−24.3(58.2)	129.9 (70.5)	−16.9 (69.1)	0.26
Fasting plasma glucose, mg/dl (SD)	102.0 (18.8)	−1.1 (14.5)	100.6 (15.9)	−3.1 (11.0)	0.29	102.5 (14.9)	−0.9(10.3)	100.4 (13.9)	−2.7 (13.8)	0.34
HbA1c (JDS),% (SD)	5.2 (0.6)	0.04 (0.36)	5.2 (0.67)	0.07 (0.38)	0.56	5.2 (0.5)	0.06(0.28)	5.2 (0.59)	0.04 (0.48)	0.75

Abbreviations: BMI, body mass index; TG, triglyceride; JDS, Japan Diabetes Society.

* Adjusted for age, height, weight, waist circumference, BMI, systolic blood pressure, diastolic blood pressure, HbA1c, total cholesterol, HDL cholesterol, LDL cholesterol, triglyceride, fasting plasma glucose at the baseline, number of the risk factors for metabolic syndrome, categories of the health counseling at baseline.

As for metabolic biomarkers, non-HDL cholesterol and TG were markedly reduced by 8.8 mg/dl and 27.0 mg/dl on average one year after the program and well preserved thereafter in the intensive care group. The usual care group members experienced a gradual reduction, but the intergroup differences for non-HDL cholesterol and TG were statistically insignificant and attenuated thereafter. Similarly, differences in HbA1c between the groups were reduced as time progressed. SBP was more decreased in the intensive care group than in the usual care group, both one and two years after the program (−3.9 vs. 1.4 mm Hg and −4.5 vs. −2.0 mm Hg, respectively), but the intergroup difference diminished after three years.

**Fig. 1:**
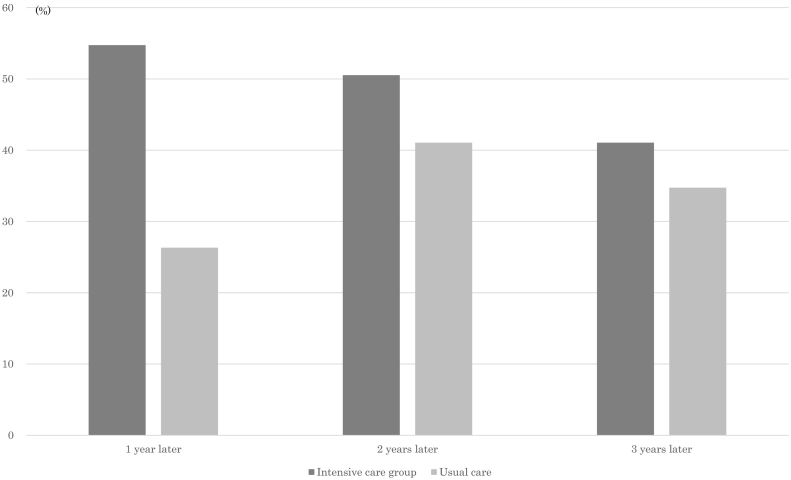
Proportions of the participants who achieved 3% reduction in body weight in 1–3 years in the propensity matched intensive care group and usual care group.

Finally, we evaluated the proportion of the subjects who achieved 3% reduction in body weight (Fig. 1). More than half of the intensive care group members attained the goal one year later, whereas only a quarter of the usual care group members did it. The number of subjects that attained 3% reduction in body weight increased thereafter in the usual care group, and the differences between the groups decreased as time progressed.

## 4 Discussion

This is the first study to report the time-dependent effectiveness of a single short-term retreat type health education session for cardiovascular risk factors. Among the employees with cardiovascular risk factors, body weight and waist circumference were significantly or marginally significantly improved after one and two years for those who underwent the three-day healthcare retreat program compared with those receiving the usual brochure-based advice. However, the intergroup differences were gradually for subsequent two years.

In this study, the mean weight reduction was 2.7 kg and 55% of intensive care participants achieved >3% weight reduction one year later. Muramoto et al. revealed that 3% weight reduction is the minimum requirement to reduce the risk of obesity-related diseases for the Japanese population (Muramoto et al., 2014). Additionally, the Japan Society for the Study of Obesity states a 3 kg body weight or 3 cm ventral girth reduction is necessary to reduce the risk of metabolic syndrome in its 2016 guidelines. Therefore, on the whole, the intervention was successful in achieving a critical reduction in body weight.

In concordance with the changes in body weight and waist circumference, serum non-HDL cholesterol and TG levels were also more reduced in the intensive care group than the usual care group, even though the standard deviations were large and the intergroup difference was statistically insignificant. Waist circumference was also changed by the intensive care. Statistical insignificance would be explained by their substantial food intake-dependent fluctuations of these items.

The findings, taken as a whole, indicate that this intervention generated improvements in several cardiovascular risk factors, without any additional organized interventions, lasting up to two years. This indicates that the program made strong and rather long-lasting impacts on the attendees willing to modify their lifestyles. The practical experience of a diet using low calorie food and simple home-based exercise without special equipment guarantees ease, simplicity, and continuity. A well-designed program built on the trans-theoretical model (Prochaska and Velicer, 1997) is another plausible explanation for the strong and long-term effects. This could program can achieve progress in the stages of health behavior based on the trans-theoretical model; such as from “contemplation stage” to “preparation stage” by reviewing the health check-up results and from “preparation stage” to “action stage” by trying low caloric meals and physical exercise. Moreover, Self-disclosure plays a central role in the development and maintenance of one's improvement (Collins and Miller, 1994; Chaudoir and Fisher, 2010), because one does make an effort when he or she was looked at by others. The overnight communal living and group work could also induce self-disclosure and solidarity among the attendees. These group dynamics of social and peer support may let the participants attain and keep their goals (Imanaka et al., 2013).

Because lifestyle modification can reduce visceral fats, lifestyle modification is the primary strategy for metabolic syndrome (Ryo et al., 2011; Takahara and Shimomura, 2014). For the prevention of diabetes mellitus, lifestyle modification was more effective than traditional pharmaceuticals (Knowler et al., 2002) and had a sustainable effect for several years (Haw et al., 2017). Moreover, Leblanc et al. reported behavior-based treatments to be safe and effective for weight loss and its maintenance in their systematic review (Leblanc et al., 2011). Our practical approach might supersede the conventional drug treatments.

The weight loss effects of this program were slightly attenuated three years later. Over time, some program attendees might abandon their acquired behaviors during the long self-care period. Refresher counseling and training within three years would be necessary to maintain success. Some previous papers discussed the necessity of maintenance interventions (Munakata et al., 2011; Moriguchi et al., 2007). For example, Moriguchi et al. showed the effectiveness of repeated counseling of 3-year interval (Moriguchi et al., 2007). In our experience, refresher training was effective, even if it was much shorter in timeframe (Nishiyama et al., 2015). Future studies showed include the refresher training within three years for the participants in the intensive program.

Of note, the employees who did not participate in the program attained some improvement in body weight, waist circumference, non-HDL cholesterol, TG, and SBP during the 3- year follow-up. Even passive yearly guidance using a brochure would prompt employees with cardiovascular risk factors to do some actions and influence their data to some extent. As such, intergroup differences were reduced within three years and the superiority of the program appears diminished, even though the post-intervention rebound was small.

This study has some inherent limitations. First, we evaluated the effectiveness based on anthropometric measurements and laboratory data. Our true endpoint is the reduction in mortality and morbidity from cardiovascular diseases. However, it is well known that the improvement of risk factors can reduce the incidence of cardiovascular diseases, and a study using surrogate markers would be of much help to daily health care for apparently healthy persons. Second, the intervention group and the control group of this study could be unequal to each other unlike a randomized controlled trial even though the propensity score matching was applied for the comparison. Nevertheless, randomized trials would evoke special attention in the participants and the study results would be shifted to the better in both intervention and control groups especially in behavior studies. Third, the findings of this study were derived from only males. Females comprise a relatively small number of this population, and age distribution is left-skewed for this company group, which is similar to the general Japanese working population. Since behaviors and risk factor profiles differ greatly by gender, further studies are warranted to elucidate the effectiveness among females. Fourth, this study was carried out in a single workplace. Then, the findings of this study cannot be universally generalized. The study results should be validated in other studies in different settings.

## 5 Conclusions

Our healthcare retreat program would improve body weight and waist circumference for two subsequent years and was worth spreading in the working population. And a three-year later refresher training would be recommended.
